# Novel Insights into *Corema album* Berries: Vibrational Profile and Biological Activity

**DOI:** 10.3390/plants10091761

**Published:** 2021-08-25

**Authors:** Joana Marques, Daniel Martin, Ana M. Amado, Viktoriya Lysenko, Nádia Osório, Luís A. E. Batista de Carvalho, Maria Paula M. Marques, Maria João Barroca, Aida Moreira da Silva

**Affiliations:** 1 Unidade de I&D Química-Física Molecular, Department of Chemistry, University of Coimbra, 3004-535 Coimbra, Portugal; dmfernandez@uc.pt (D.M.); ama@uc.pt (A.M.A.); nadia.osorio@estescoimbra.pt (N.O.); labc@ci.uc.pt (L.A.E.B.d.C.); pmc@ci.uc.pt (M.P.M.M.); mjbarroca@esac.pt (M.J.B.); aidams@esac.pt (A.M.d.S.); 2 College of Health Technology of Coimbra, Polytechnic Institute of Coimbra, S. Martinho do Bispo, 3046-854 Coimbra, Portugal; v.lysenko2@health.gov.je; 3 Department of Life Sciences, University of Coimbra, 3000-456 Coimbra, Portugal; 4 Polytechnic of Coimbra, Coimbra Agriculture School, Bencanta, 3045-601 Coimbra, Portugal

**Keywords:** white crowberries, plant extracts, antibacterial activity, nutraceutical, FTIR and Raman spectroscopies

## Abstract

This study reports an evaluation of the biological properties of the edible berries from *Corema album*, an endemic shrub of the Portuguese coastline, aiming at its use as a nutraceutical. Different methanolic extracts were obtained from the pulp and seed of fresh berries: pulp extract, seed residue, and seed oil (extracted and characterized for the first time). For each of these, the antioxidant activity was assessed, by different methods, as well as the antimicrobial ability. Overall, the seeds were shown to be the most nutraceutical part of the berry since they showed higher antioxidant activity, while the pulp extract displayed a significant antimicrobial capacity against several clinically relevant bacterial strains. Furthermore, the extracts were fully characterized by complementary infrared and Raman spectroscopy, revealing the presence of phenolic acids, polysaccharides, sugars, and triterpenoids in the pulp, high content of unsaturated fatty acids in the seed oil, and significant amounts of phenolics and carotenoids in the seed residue. These results pave the way for a reliable correlation between chemical composition and biological activity, in edible fruit samples.

## 1. Introduction

The Portuguese coastline is rich in many indigenous maritime plants with a high potential to become novel functional food ingredients (or sources of these). *Corema album* (L.) D. Don is a dioecious perennial shrub of the Ericaceae family, endemic of the Iberian Peninsula Atlantic coastal dunes. It is a branched bush, that can reach up to 1 m, with white acidic edible berries (Portuguese white crowberries or “camarinhas” in Portuguese), 5–8 mm in diameter [1]. The genus *Corema* was included in the Ericaceae family in 1959 since traditionally it belonged to the Empetraceae family, which comprises two more genus—*Empetrum* and *Ceratiola*. The two species of *Corema* genus, *C. conradii* Torrey and *C. album* (L.) D. Don ex Steudel, can be found in Atlantic coastlines—*C. conradii* in the eastern coast of North America and *C. album* in the Iberian Peninsula and in the Azores islands (subsp. *azoricum* Pinto da Silva) [2].

*C. album* berries are known to be exceptional sources of nutrients and phytochemicals. Their dietary intake is highly recommended since it is associated with the prevention of chronic and degenerative diseases [3]. The nutraceutical potential of berries is due to their phytochemical composition. High levels of phenolic compounds have been identified, particularly phenolic acids (benzoic and caffeic acids being the most predominant), flavonols (especially quercetin 3-*O*-hexoside and rutin), anthocyanins (delphinidin 3-*O*-hexoside, cyanidin 3-*O*-glucoside, and cyanidin 3-*O*-pentoside), and tannins [4,5,6]. This confers them beneficial biological properties, namely antioxidant, antimicrobial, and anticancer activities, rendering them promising chemopreventive agents against anti-inflammatory and anti-neurodegenerative disorders, as well as cancer [7,8]. The berries have also been described as a valuable source of fibers, water, and sugars [9].

The human consumption of *C. album* berries dates back to ancient times, having been used in traditional medicine as antipyretic [10] and antiparasitic agents [11]. Since they are not yet approved as novel food products—having not entered into the regular market—these types of berries are not consumed by the general population [12]. However, they have been sold in Portuguese local markets, in the regions where *Corema* exists. Even though this plant has gained the attention of the scientific community in the last few years, the number of published studies, at the molecular level, concerning the biological potential of *C. album* berries and their nutritional value is still scarce, e.g., less than 10 papers, using Science Direct and Scopus digital databases by searching for specific keywords within the title (“*Corema album*”) and (“antioxidant”) in abstract. A thorough characterization of these berries is essential for understanding their activity, as well as to allow their safe consumption either as fresh fruits or processed in the form of juices, jams, or jellies. Martin et al. (2020) reported the first spectroscopic study of fresh *C. album* berries, assigning distinct vibrational fingerprints to the skin and the seeds that revealed the differences in their content in phenolic derivatives, unsaturated fatty acids, and waxy polymers [13].

Since the evaluation of the biological properties of the different parts of *C. album* berries, as well as their spectroscopic characterization, is still scarce, the present study aims at filling this gap, particularly for extracts from fresh pulp, seed residue, and seed oil. Actually, only two studies are found in the literature for *C. album* pulp and seed [4,14], with a small number of antioxidant activity tests, without any vibrational spectroscopic characterization, and with no separation between the seed residue and the seed oil. Solvent extraction with methanol was the method of choice since this combination is routinely used for phytochemicals extraction with good yields [15]. Currently, antioxidant activity was measured for isolated extracts from the pulp (also tested for antimicrobial activity), seed residue, and seed oil, and the results were related to the main chemical constituents determined by both Fourier transform infrared (FTIR) and Raman vibrational spectroscopies. Apart from probing its separated constituents, the establishment of a relationship between composition and health beneficial effects is innovative for this edible fruit.

## 2. Results

### 2.1. Total Phenolic, Flavonoid, and Monomeric Anthocyanin Content

The fresh berries pulp (FBP) extract presents the lower phenolic, flavonoid, and anthocyanin contents when compared to the berries seed residue (BSR) and berries seed oil (BSO) extracts (Table 1). The results show that the seeds are much richer in phenolic and flavonoid compounds and that the reddish BSR extract has the highest total monomeric anthocyanin content (TMAC) value.

### 2.2. Antioxidant Activity

The BSR extract presents a higher radical scavenging ability both against the DPPH radical and the ABTS radical cation (Table 2), followed by the FBP extract and by the BSO, which presents the lowest antioxidant activity. Noteworthy is the EC_50_ value for the BSR in the DPPH assay, which is in the same range as the EC_50_ calculated for BHT. Moreover, only the BSR extract presents the capability to inhibit lipid peroxidation, though to a lower extent than the standard antioxidant BHT.

**Figure 1:**
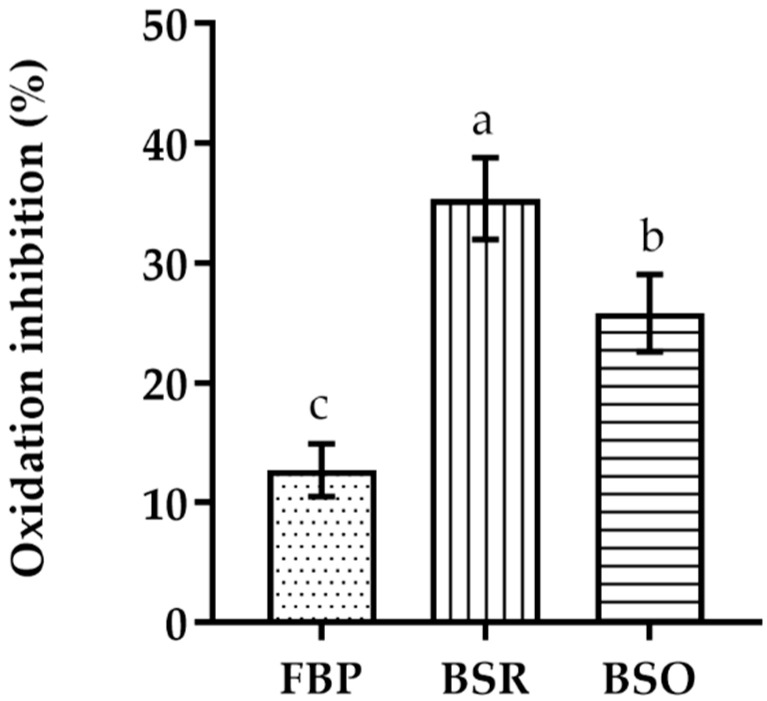
Linoleic acid/β-carotene bleaching inhibitory activity of extracts of different parts of *C. album* berries. FBP, fresh berries pulp (dotted); BSR, berries seed residue (vertical bar); BSO, berries seed oil (horizontal bar). Values represent the mean ± standard deviation of three independent experiments obtained after 2 h of reaction and for the highest concentration of each extract. Bars with different lowercase letters (a–c) indicate significant differences (Tukey’s post hoc test, *p* < 0.05).

In the β-carotene–linoleic acid bleaching assay, the BSR extract presents a higher inhibition of the β-carotene oxidation than the other extracts (Figure 1). Nevertheless, it is still considerably less active than BHT (EC_50_ = 0.005 ± 0.002 mg/mL). The BSO extract showed a higher level of β-carotene oxidation inhibition when compared to the FBP extract.

Regarding the metal ion chelator ability, the results presented in Table 3 reflect the trend already observed for the BSR extract. It shows to be more potent than the other analyzed extracts regarding the ferric and cupric reducing powers, as well as the ability to chelate iron, though to a lower extent than the EDTA chelator. All the extracts are more effective in reducing copper than iron, with FRAP values ranging from 6.8 to 54.7 mg TE/g extract and CUPRAC values in the 24.7–146.6 mg TE/g extract range. 

### 2.3. Enzyme Inhibitory Effect

Acetylcholinesterase inhibition was tested for the FBP, BSR, and BSO extracts, but no significant activity was observed in the range of concentrations tested. 

### 2.4. Antimicrobial Activity

The FBP extract was tested regarding its ability to reduce bacterial growth or even induce bacterial death. The results thus gathered (Table 4) show significant antimicrobial activity against several bacterial strains with clinical importance, displaying different antibiotic susceptibility profiles (from more susceptible to more resistant ones), ranging from a MIC value of 3.125 to 50 mg/mL.

The antimicrobial activity presently measured was found not to be related to the antimicrobial susceptibility phenotype, since the MIC value obtained against *Staphylococcus aureus*, for instance, was 12.5 mg/mL both in the ATCC 29213 sensitive strain and in the resistant MRSA.

### 2.5. Spectroscopic Analysis

**Figure 2:**
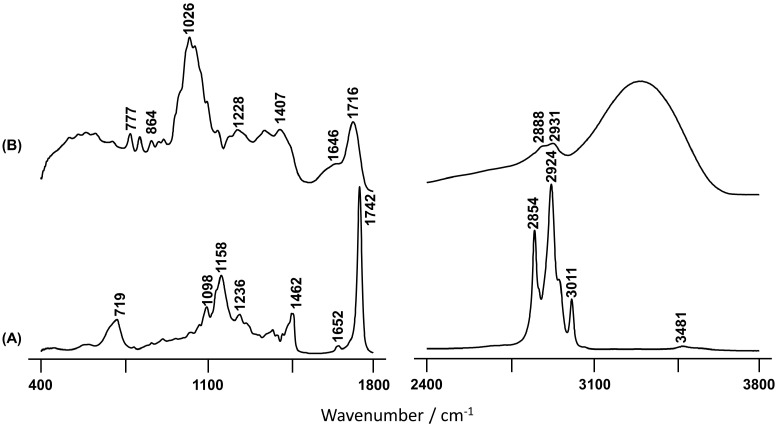
FTIR-ATR spectra of the BSO (**A**) and FBP (**B**) *C. album* berry extracts.

Figure 2 comprises the FTIR-ATR spectra of the BSO (2A) and FBP (2B) extracts, in the 400–1800 cm^−1^ and 2400–3800 cm^−1^ regions, the most prominent bands being highlighted. Significant differences between both spectra were detected (see Discussion).

**Figure 3:**
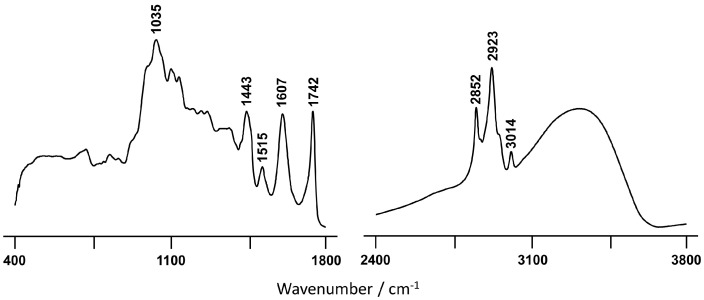
FTIR-ATR spectrum of the BSR *C. album* berry extract.

Figure 3 depicts the ATR-FTIR spectrum of the BSR extract from the *C. album* seeds in the 400–1800 cm^−1^ and 2400–3800 cm^−1^ regions.

**Figure 4:**
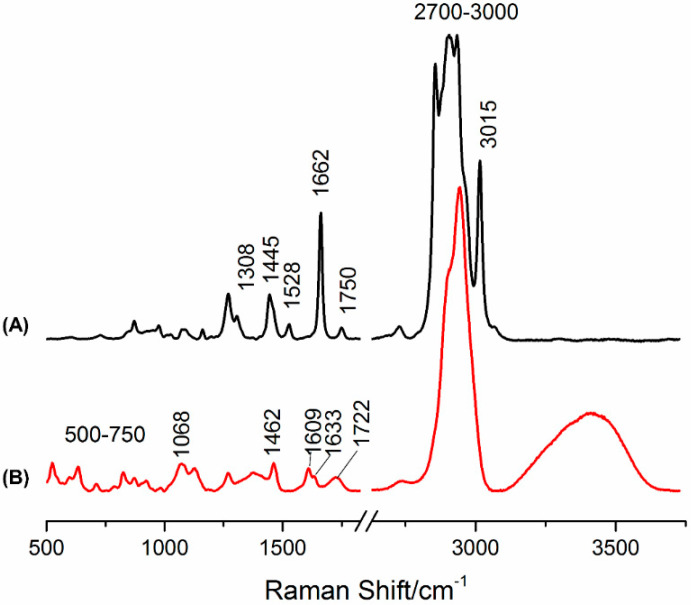
Raman spectra of the BSO (**A**) and FBP (**B**) *C. album* berry extracts.

Figure 4 shows the Raman spectra of the BSO and FBP extracts in the spectral region 400–3750 cm^−1^. Regarding BSO, the similarities with the data obtained by Martin et al. (2020) for the pure sample are remarkable. One of these concerns the signal at 1528 cm^−1^, which is undetected in the pure seed. This band is assigned to ν(C=C) found in conjugated polyenes such as lutein (widely present in oilseeds of many plants) [13,16]. Additionally, it is noteworthy that the signal at 3015 cm^−1^ (Figure 4A), which evidences that the fatty acids/esters within this sample are *cis*-saturated. The ν(C=O) feature of these components is also observed at 1750 cm^−1^. Finally, the signal at 1662 cm^−1^ is also ascribed to the ν(C=C) mode from unsaturated fatty acids. 

Regarding Figure 4B, the range between 500–750 cm^−1^ is highly remarkable, comprising bands from major components such as sugars and triterpenoids (the latter mainly from the skin of the berry). Additionally, the signals at 1060–1090 cm^−1^ may be assigned to a glycosidic linkage, reflecting a high number of polysaccharides in the sample.

One of the main aspects of the Raman profile is the bands found at 1609 (ν(C=C)_ring/propenoic chain_) and 1633 cm^−1^ (ν(C=O)_ring, chain_), which corroborate the presence of high amounts of phenolic acids. In fact, these signals are characteristic of ferulic and *p*-coumaric acids [17,18,19,20] and are complemented with the feature at 1722 cm^−1^ ascribed to the ν(C=O) mode from phenolic acid derivatives [13]. As expected, the water amount is clearly higher for the FBP extract (Figure 4B) as compared to BSO (Figure 4A).

Regarding the BSR extract, the most striking point regarding its Raman profile (Figure S1 of Supplementary Material) is the large number of carotenoids, which is higher than that detected for the BSO sample.

## 3. Discussion

A plethora of methods are described and accepted for the determination of the antioxidant potential of plants, comprising the measurement of the phytochemical composition and different molecular reactions such as radical scavenging, metal chelation, and reducing power. For this study, several of these methodologies were chosen, ranging from the evaluation of free radical scavenging and lipid peroxidation inhibition to metal chelation/reduction potentials and enzymatic inhibitory activity [21]. The synthetic antioxidant BHT, commonly employed as a food preservative, was used as a model antioxidant for comparison purposes [22].

The berries show great complexity and diversity of phytochemicals, namely phenolic acids (in both their free, ester, and glycosidic forms), flavonoids, and tannins, among others [23]. Acetone extracts of *C. album* berries revealed the presence of this wide variety of compounds: 77.5% phenolic acids, 21.8% flavonoids, and 0.66% anthocyanins [5]. As for other berries of the genera *Vaccinium, Sorbus*, *Empetrum,* or *Sambucus*, the hydroxycinnamate chlorogenic acid is the main phenolic acid found in the *C. album* berries [5,24]. However, as the berries have a high proportion of seeds (54.9% of dry weight) with different compositions relative to the other parts of the fruit, the analysis of these separate extracts is particularly relevant. In fact, from the published studies on this type of berry, only two [4,14] focused on pulp and seeds, while all the others studied the fruit as a whole.

In the present study, it was found that the seeds are richer in phenolic and flavonoid compounds, which contrasts with previous data obtained for methanolic extracts of the pulp and seed of freeze-dried white *C. album* berries [4]. However, the use of different matrices (fresh pulp in the present study versus freeze-dried pulp extracts) might explain these differences. Nevertheless, extraction of the seed components was still more efficient, though different extraction methods were applied, namely magnetic stirring (room temperature, 1 h) versus ultrasonic bath (40 °C, 1 h), both with methanol as the solvent [4]. Nonetheless, acetone/water extracts of dehydrated pulp and seeds confirmed the higher TPC of the seeds [14].

The major flavonoids compounds identified in Portuguese crowberry fruits are quercetin followed by rutin [5] that are compounds with great antioxidant activity, as evidenced by the Trolox equivalent antioxidant capacity (TEAC) of 4.72 and 2.4 mM, respectively [25]. In fact, flavonoids are phenolic plant metabolites that play an antioxidant effect, but recently there is evidence that the most abundant flavonoids present in the vegetable matrix have a dual behavior [26,27,28]. Indeed, these antioxidant compounds can act as a prooxidant, inducing oxidative stress under certain conditions such as the concentration of the antioxidant in the matrix, the presence of metal ions and its redox potential [29,30,31]. Specifically, low molecular weight phenolic molecules such as quercetin and gallic acid, which are easily oxidized, have a known pro-oxidant activity [32]. The results suggest that some flavonoid compounds present in the BSO extract, as well as their concentration, can induce a prooxidant behavior of the extract. Additionally, the predominant subclasses of flavonoids present in BSO extract can be flavone and flavanone since they have no -OH substitutions that are required for antioxidant activity [33].

The extracts were obtained from ripe *C. album* white berries, already described as presenting only small amounts of anthocyanins, which agrees with the lower TMAC content (Table 1) found in the FBP extract and the lower TPC when compared to other colored berries [5]. Nevertheless, the reddish BSR extract has the highest TMAC value, indicating that this part of the berry concentrates more anthocyanins, which may contribute to its higher TPC in comparison with the other extracts. Despite the low content in anthocyanins of this wild *C. album* berry, their large amount of total phenolic compounds and high antioxidant capacity are at a similar level to strawberry tree fruit and raspberries [34].

Among the three extracts tested, and in agreement with the higher content in total phenolics and anthocyanins, which would confer an enhanced antioxidant activity, the BSR extract presents a higher radical scavenging ability (Table 2) both against the DPPH and the ABTS free radicals. The presence of esters in the berry seeds, clearly detected by FTIR-ATR (Figure 3), may indicate that the hydroxycinnamate chlorogenic acid, which is an efficient antioxidant agent [35,36,37], is mainly concentrated in the seeds. The DPPH radical scavenging activity of the BSR extract (EC_50_ = 0.15 mg/mL) was found to surpass that obtained for *Citrus sinensis* seeds (in both hydroethanolic and ethanolic extracts, EC_50_ = 0.18 and 0.34 mg/mL, respectively) [38]. Although BSO displays the highest flavonoid content, it has the lowest antioxidant activity. However, the antioxidant activity of phenolic compounds such as flavonoids significantly depends on the structure and concentration of the molecules in the extract, since intermolecular interactions are prone to affect the redox profile of these antioxidants. Moreover, only the BSR extract presented the capacity to inhibit lipid peroxidation (Table 2), though to a much lower extent than the standard antioxidant BHT, which could be explained by the higher content in polyphenolic compounds in this extract. These results are comparable to those previously reported for black raspberry seed residues extracts (after oil extraction) with EC_50_ values ranging from 1.132 to 1.255 mg/mL [39].

Regarding the β-carotene–linoleic acid bleaching assay (Figure 1), as well as the metal chelating ability and reducing powers (Table 3), the BSR extract presented the highest inhibition of oxidation of β-carotene coupled to a stronger ability to chelate and reduce metals. This is in accordance with the results currently obtained in the other antioxidant tests. Actually, the phenolic compounds in this extract are suggested to be mainly molecules such as hydroxyflavones (e.g., quercetin and rutin) which have the ability to alter their redox potential, thus becoming very effective chelating agents to potentially oxidative metal ions (such as Fe^3+^ and Cu^2+^) [25,40]. Therefore, this type of phenolics will exert their antioxidant activity in a twofold manner. This effect is even more noteworthy in the β-carotene–linoleic acid bleaching assay since this extract was tested at a concentration of 4 mg/mL while the other extracts were used at 5 mg/mL. It is interesting to note that the BSO extract was much more efficient in reducing copper than the FBP extract, which contrasts with their behavior regarding the ferric reducing power. Actually, the BSO extract only presents relevant activities in the CUPRAC and β-carotene–linoleic acid bleaching assays, being almost as potent as the BSR extract.

Natural products have been the source of many new drugs (namely against cancer and neurodegenerative disorders), over 119 natural molecules have been shown to be efficient acetylcholinesterase inhibitors, one of the currently available treatment options for Alzheimer’s disease [41]. This prompted the evaluation of the inhibitory activity of the *C. album* extracts against the enzyme AChE, though no significant effect was observed for any of the samples tested.

Vibrational spectroscopy techniques have been recognized as powerful tools for identifying functional groups in diverse types of samples, including heterogeneous biological matrices, from a qualitative and semi-quantitative point of view. Thus, it has been largely employed in plant science in the last decades, specifically for the study of edible oils such as olive oil, or unsaturated fatty acids [42,43,44,45,46,47,48]. Additionally, these methods have also been applied routinely to the analysis of phenolic compounds [49,50,51].

While it is true that HPLC combined with mass spectrometry is a suitable and very commonly used technique for identifying the main constituents of these types of plant extracts, vibrational spectroscopy (e.g., FTIR and Raman) is able to provide highly accurate chemical data, with unmatched sensitivity and specificity, in a fast and completely non-destructive way, while requiring minimal amounts of extract and no sample preparation. Hence, FTIR is currently a method of choice for evaluating the chemical composition (major constituents) of several types of biological extracts, used routinely in the food industry.

The FTIR-ATR spectrum obtained for the BSO extract from the *C. album* (Figure 2A) presents one prominent band observed at 3011 cm^−1^, which was assigned to olefinic *cis*-unsaturation in fatty acids that are known to be present in this kind of edible fruit samples [46,52]. Thus, this intense band suggests a high content of unsaturated fatty acids in BSO, which is also supported by the observation of a signal at 1652 cm^−1^ from the C=C stretching mode of *cis* fatty acids [46]. On the other hand, the feature at 1742 cm^−1^, characteristic of esterified fatty acids [53], reveals a considerable amount of esters attending to the quite large shift relative to ν(C=O)_acid_ [54]. The Raman spectrum of the BSO is very similar to that of the pure seed previously reported [13], thus showing that the main seed components are those present in the oil. In the light of these results, it is possible to conclude that the efficiency of the extraction procedure currently performed was quantitative, constituting an interesting methodology for future studies of edible fruits.

Regarding the infrared spectrum of the FBP extract (Figure 2B), the results reflect a high content of hydroxylic components, namely phenolic acids. This is clearly evidenced by the broad and intense band centered at 3303 cm^−1^ and ascribed to the stretching mode from the ring hydroxyls. Additionally, the signal at 777 cm^−1^ is characteristic of out-of-plane (C-C-H) deformation modes of the aromatic ring [49]. This type of compound seems to be absent in the oil extract, as evidenced by the total absence of vibrational bands in the high wavenumber spectral region (Figure 2A). It should be highlighted that the spectrum of the seeds revealed the presence of a significant amount of hydroxylic compounds, as well as water [13], which were most likely removed during the oil extraction process. The broad infrared signal at 1026 cm^−1^, which is absent in BSO, is due to the ν(C-O) and ν(C-C) modes from polysaccharides and pectins, that are widely present in these types of samples [55,56,57,58]. As expected, it is more likely to find sugars in the fresh pulp in comparison with the oleaginous seed part, which is clearly evidenced when BSO and FBP spectra are compared (Figure 2).

The BSR fraction still contains a significant amount of esterified fatty acids, as evidenced by the infrared spectral features at 3014 and 1742 cm^−1^ (Figure 3). However, probably the most remarkable characteristic, in contrast to BSO (Figure 2A) is that the infrared profile of BSR shows several features indicative of significant amounts of phenolic compounds, namely three very distinctive bands at 1443, 1515 cm^−1^, and 1607 cm^−1^. The first two are ascribed to ring ν(C-C) conjugated with (C=C), while the last one is assigned to ν(C-C)_aromatic_ [59,60,61]. Furthermore, the presence of more carotenoids in BSR relative to the other extracts, detected by Raman (Figure S1), may contribute to the high antioxidant activity currently determined for this sample. Although Raman spectroscopy does not allow an accurate carotenoid quantification in the samples, it does enable us to determine, in a semi-quantitative manner, in which sample this polyene is present on the highest content. This was carried out by comparing the peak ratio I_1528_/I_1448_ [13], which reaches a value higher than 1 for BSR, being below unity in the case of BSO. These observations are in good accordance with the higher antioxidant potential determined for the BSR extract relative to the one obtained for BSO.

In the present study, the spectroscopic characterization of the extracts by FTIR-ATR and Raman allowed the detection of fatty acids in the BSO and BSR, and of phenolic compounds in FBP and BSR. The FBP extract was also found to contain sugars, triterpenoids, and polysaccharides. In addition, the presence of glycosidic linkages may also indicate that most of the phenolic acids are conjugated to sugar moieties. In fact, these chemical characteristics evidence the complexity of the sugar polymers present in the sample [62,63].

Finally, it may be interesting to compare the presently obtained results with those from studies previously performed by other authors on *C. album*. In particular, León-González et al. [7] analyzed the phenolic content of the berries using different extraction methodologies. A large number of phenolic acids was identified by these authors (by HPLC and MS), in some cases reaching ca. 2260 mg per kg of extract [5]. Despite the fact that the solvents were different from the ones used in the current study, the main extracted compounds were found to be the same.

Since *C. album* has been used by some ancient civilizations to eliminate intestinal worms [1], the present study aimed to evaluate the antimicrobial activity of the FBP sample, since certain antibiotics are also used to treat intestinal parasites (e.g., metronidazole) [64]. Comparison of the current results with the few studies in the literature describing the evaluation of antimicrobial activity for plant extracts of the same taxonomic class allows us to conclude that the FBP extract of *C. album* displays promising antibacterial activity: a MIC = 17 mg/mL for extracts of *Lythrum salicaria* against *Pseudomonas aeruginosa* [65] relative to a MIC = 12.5 mg/mL (Table 4), and the lowest activity of *Tamarix gallica* extracts observed against *Escherichia coli* using disk diffusion method [66], relative to a MIC = 6.25 mg/mL (Table 4), which contrasts with the lack of antibacterial activity obtained for acetone/water extracts of *C. album* pulp testing lower concentrations and using the disk diffusion method [14]. The inhibitory effect mechanism of FBP against the several strains currently tested is not known, however, based on some known compounds that may be present namely hydroxycinnamic acids, vanillic acid, and quercetin [5], could be related to their antioxidant mechanisms. Studies on the antimicrobial activity of these phenolic compounds and isolated flavonoids, showed significant antimicrobial activity against *E. coli, P. aeruginosa, K. pneumoniae,* and *S. aureus*, among others [67]. The recognized inhibitory effect of phenolic compounds towards bacterial growth can be explained by their ability to increase the cell membrane permeability, this effect varying for different bacterial strains due to differences in their structure and lipophilic character [68]. It may also be due to their ability to adsorb to cell membranes, interact with enzymes and other biological substrates, sequester metal ions that, in general, affect the normal cellular function [67]. In future work, it will be relevant to understand which components within the FBP extract are responsible for the measured antimicrobial effect and how they can lead to cellular damage in different types of microorganisms.

## 4. Materials and Methods

### 4.1. Chemicals 

2,2′-Azino-bis(3-ethylbenzothiazoline-6-sulphonic acid) diammonium salt (≥98%), 2,2′-azobis(2-methylpropionamidine) dihydrochloride (97%), 2,2-diphenyl-1-picrylhydrazyl, 2,4,6-tris(2-pyridyl)-*s*-triazine (≥99%), 5,5′-dithiobis(2-nitrobenzoic acid) (99%), acetylcholinesterase (200–1000 units/mg protein) from *Electrophorus electricus* (electric eel), acetylthiocholine iodide (≥99.0%), aluminum chloride (AlCl_3_, for synthesis), ammonium acetate (≥98%), butylated hydroxytoluene (≥99%), copper(II) chloride (CuCl_2_, for synthesis), ethylenediaminetetraacetic acid (≥98.5%), ferrozine (97%), galantamine hydrobromide, gallic acid (≥98.0%), iron(II) chloride (FeCl_2_·4(H_2_O), ≥99%), linoleic acid (≥99%), Mueller Hinton Broth, neocuproine (≥98%), *p*-Iodonitrotetrazolium Violet, potassium chloride (≥99%), quercetin (≥95%), sodium acetate (≥99%), sodium carbonate (≥99.5%), sodium dihydrogen phosphate (NaH_2_PO_4_·2(H_2_O), ≥98%), thiobarbituric acid (≥98%), trichloroacetic acid, TRIS (≥99%), Trolox (97%), Tryptic Soy Agar, Tween^®^ 80, β-carotene (≥93%), as well as solvents (of analytical grade) were obtained from Merck (Oeiras, Portugal). Acetic acid (glacial p.a.) was purchased from Pronalab (Sintra, Portugal), the Folin-Ciocalteu’s reagent, HCl (35%) and iron(III) chloride (FeCl_3_·6(H_2_O)) (≥98%) from Panreac (Barcelona, Spain), and potassium persulfate (99%) and sodium phosphate dibasic (Na_2_HPO_4_, ≥99%) from Honeywell (Carnaxide, Portugal).

### 4.2. Plant Samples

Wild *C. album* berries (5 kg) were harvested by hand in early September 2019, randomly selected from 20 independent bushes, in the dunes of the Quiaios Beach (Figueira da Foz) in the central region of Portugal (40°13′15.8″ N 8°53′24.9″ W). Ripe white berries (spherical, 0.3–0.5 g, with a diameter of ~5–8 mm) were collected and immediately prepared according to each extraction procedure.

### 4.3. Extraction Procedures

Two extractions were performed using different parts of the berries: fresh berry pulp (FBP) and dried berry seeds. The fresh pulp (skin included) was separated from the seed and crushed, the fresh seeds were dried at 40 °C for 24 h and ground. The samples were extracted immediately, in a ratio of 1:20 (*w*/*v*) with methanol, for 1 h, at room temperature, in a magnetic stirrer. Upon centrifugation at 1018× *g* for 10 min, the supernatant was filtered and evaporated under vacuum (in an R-200 rotary evaporator, Buchi, Switzerland). The FBP extract was freeze-dried and stored at room temperature (extraction yield of 8.2 g/100 g fresh pulp). The evaporation of the seed extract yielded two fractions, a residue (BSR) and an oil (BSO), which were separated by decantation. The BSR extract was freeze-dried and stored at room temperature, while the BSO was stored at 4 °C (extraction yields of 2.7 g/100 g and 1.8 mL/100 g dried seeds, respectively). Stock solutions were prepared and stored at 4 °C prior to the determination of chemical composition and evaluation of antioxidant potential. The FBP and BSO extracts were solubilized in ethanol at a concentration of 5 mg/mL, while for BSR, methanol was used, allowing to reach a concentration of 4 mg/mL. 

### 4.4. Total Phenolic Content

The total phenolic content (TPC) was determined using the modified version of the Folin-Ciocalteu method previously reported [69]. Briefly, 50 μL of Folin-Ciocalteu (50%, *v*/*v*) reagent and 400 μL of Na_2_CO_3_ (5%, *w*/*v*) were added to 50 μL of the test sample, in a 48-well plate, in triplicate. The plate was shaken, incubated at room temperature for 20 min (in the dark) and the absorbance was measured at 760 nm (in a µQuant™ Microplate spectrophotometer, BioTek Instruments Inc., Bad Friedrichshall, Germany), against a blank consisting of a mixture of the reagent’s solvents. In addition, gallic acid (GA) was used as a reference standard (in the 5–60 μg/mL concentration range) and the TPC was expressed as mean ± standard deviation of three independent experiments as equivalents of gallic acid per gram of extract (mg GAE/g extract).

The above-described procedure was also applied to the BSO extract, following the methodology reported by other authors [70] which enables to directly quantify the oil sample after solubilization in an appropriate solvent, without previous extraction of the phenolic compounds. The currently prepared ethanolic solution of the seed oil was easily mixed with the aqueous Folin-Ciocalteau reagent, with no precipitates having been formed, and thus allowing accurate spectrophotometric readings.

### 4.5. Total Flavonoid Content

The total flavonoid content (TFC) was determined using the method previously described in the literature [71], with slight adaptations. Briefly, 100 μL of AlCl_3_ (1%, *w*/*v*) were added to 100 μL of the test sample, in a 96-well plate, in triplicate. The plate was shaken, incubated for 10 min (in the dark) at room temperature and the absorbance readings were measured at 425 nm, against a blank consisting of a mixture of the reagent’s solvents. The absorbance of the extracts at 425 nm was subtracted. Quercetin (QC) was used as a reference standard (in the 7.5–75 μg/mL concentration range) and the TFC was expressed as mean ± standard deviation of three independent experiments as equivalents of quercetin per gram of extract (mg QCE/g extract).

### 4.6. Total Monomeric Anthocyanin Content

The total monomeric anthocyanin content (TMAC) was determined by the pH differential method previously reported [72]. In a 48-well plate, 80 μL of test sample were added to 320 μL of potassium chloride (pH = 1.0, 0.025 M) and sodium acetate (pH = 4.5, 0.4 M) buffers, respectively, in triplicate. The plate was shaken, incubated for 15 min (in the dark) and the absorbance was measured at 520 and 700 nm, against a blank consisting of a mixture of the reagent’s solvents.

The TMAC was expressed as mean ± standard deviation of three independent experiments as equivalents of cyanidin-3-glucoside (C3G, used as a reference) per gram of extract (mg C3GE/g extract) and calculated using (Equation (1)) (based on the Beer-Lambert law):

(1) $$\mathrm{TMAC}=\frac{A\times \mathrm{MW}\times \mathrm{DF}\times 10^{3}\;}{\varepsilon \times l}$$

where A represents the absorbance of the extracts in the two buffers ($A={\left(A_{520}-A_{700}\right)}_{\mathrm{pH}\;1.0}-{\left(A_{520}-A_{700}\right)}_{\mathrm{pH}\;4.5}$), MW is the molecular weight of C3G (442.9 g/mol), DF is the dilution factor, 10^3^ is the factor to convert g to mg, ε is the molar extinction coefficient of C3G (26,900 L/mol.cm) and l is the pathlength (cm). 

### 4.7. 2,2-Diphenyl-1-picrylhydrazyl Radical Scavenging

The ability to scavenge the 2,2-diphenyl-1-picrylhydrazyl (DPPH) radical was determined as previously described [73]. The DPPH radical was prepared in methanol in order to have a maximum absorption value at 515 nm in the 0.9–1.0 range. In a 96-well plate, 100 μL of DPPH• solution was added to 100 μL of different extract concentrations (1:2 serial dilutions from the initial sample), in triplicate. DPPH• scavenging was verified at 515 nm, after 30 min of incubation at room temperature (in the dark), against a blank consisting of a mixture of the reagent’s solvents. A mixture of DPPH• and either ethanol or methanol (depending on the extract) was used as a control and butylated hydroxytoluene (BHT) as a reference antioxidant. The percentage of DPPH• scavenging was determined for each extract using Equation (2):

(2) $$\mathrm{Antiradical}\;\mathrm{effect}\;\left(\%\right)=\frac{Abs_{control}-Abs_{sample}}{Abs_{control}}\times 100,$$

The DPPH• scavenging activity was expressed as the half-maximal effective concentration (EC_50_, mg/mL), calculated using a nonlinear regression analysis. Three independent experiments were performed and averaged.

### 4.8. 2,2′-Azino-bis(3-ethylbenzothiazoline-6-sulfonic acid) Radical Cation Scavenging

The scavenging ability towards the 2,2′-azino-bis(3-ethylbenzothiazoline-6-sulfonic acid) radical cation (ABTS•+) was determined as previously reported [74], with slight modifications. The ABTS radical cation was formed by adding 5 mL of a 4.9 mM potassium persulfate solution to 5 mL of a 14 mM ABTS solution and kept for 16 h in the dark. This solution was then diluted with ethanol to reach an absorbance value at 734 nm of 0.7, to be used in further assays. In a 96-well plate, 190 μL of ABTS•+ radical was added to 10 μL of different extract concentrations (1:2 serial dilutions from the initial sample), in triplicate. After 6 min of incubation at room temperature (in the dark), an absorbance reduction was verified at 734 nm against a blank consisting of a mixture of the reagent’s solvents. A mixture of ABTS radical cation and either ethanol or methanol (depending on the extract) was used as control and BHT as a reference antioxidant. The percentage of ABTS•+ scavenging was determined for each extract using Equation (2), and the results were expressed as EC_50_ values (mg/mL) calculated using a nonlinear regression analysis. Three independent experiments were performed and averaged.

### 4.9. β-Carotene–Linoleic Acid Bleaching Method

The inhibition of the coupled oxidation of the linoleic acid/β-carotene system was assessed using a modified version of reported methods [75,76]. Linoleic acid (60 mg) and Tween^®^80 (200 mg) were added to β-carotene (10 mg) in 10 mL chloroform in a round bottom flask, and the suspension was vigorously shaken. The chloroform was evaporated under vacuum at a temperature below 50 °C and the oily residue was diluted with bidistilled water. The resulting emulsion was vigorously shaken. Using a 24-well plate, 500 μL of emulsion were added to 1 mL of ultrapure water and 25 μL of the highest concentration of each extract (FBP—5 mg/mL, BSR—4 mg/mL, BSO—5 mg/mL), in triplicate. The absorbance was measured at 460 nm at *t* = 0 h and *t* = 2 h, after incubation at 50 °C (in the dark), against a blank consisting of a mixture of the reagent’s solvents. A mixture of the emulsion with either ethanol or methanol (depending on the extract) was used as control and BHT as a reference antioxidant. The percentage of β-carotene oxidation inhibition after 2 h was calculated using Equation (3) and expressed as mean ± standard deviation of three independent experiments.

(3) $$\mathrm{Oxidation}\;\mathrm{Inhibition}\;\left(\%\right)=\left[1-\frac{Abs_{sample}^{t=0}-Abs_{sample}^{t=2}\;\;}{Abs_{control}^{t=0}-Abs_{control}^{t=2}}\right]\times 100,$$

### 4.10. Inhibition of Lipid Peroxidation in Buffered Egg Yolk

The inhibition of the formation of thiobarbituric acid reactive substances (TBARS) was evaluated as previously reported [77]. A mixture of chicken egg yolk (500 μL, 0.1 g/mL) in phosphate buffer (0.1 M, pH = 7.4) and 50 μL of 2,2′-azobis(2-methylpropionamidine) dihydrochloride (AAPH, 0.12 M) were added to 50 μL of each extract concentration (1:2 serial dilutions from the initial sample). Each mixture was incubated at 37 °C for 1 h, treated with 250 μL of 15% (*v*/*v*) trichloroacetic acid and 500 μL of 1% (*w*/*v*) thiobarbituric acid, and kept at 95 °C for 10 min. Upon cooling, the samples were centrifuged at 3500 rpm for 10 min and the absorbance of the supernatant was measured at 532 nm, against a blank consisting of a mixture of the reagent’s solvents. A mixture of buffered egg yolk, AAPH, and either ethanol or methanol (depending on the extract) was used as the control and BHT as a reference antioxidant. The percentage of inhibition of lipid peroxidation was determined for each extract using Equation (2), and the results were expressed as EC_50_ values (mg/mL) calculated using a nonlinear regression analysis. Three independent experiments were performed and averaged. 

### 4.11. Metal Chelating Ability

The iron-chelating activity was measured as previously reported [78], with some modifications. In a 96-well plate, 50 µL FeCl_2_·4(H_2_O) (0.1 mM) and 100 µL of ferrozine (0.25 mM) were added to 50 µL of each extract concentration (1:2 serial dilutions from initial sample), in duplicate. After incubating at room temperature for 10 min (in the dark), absorbance was measured at 562 nm against a blank consisting of all the reagents except for ferrozine. A mixture of all the reagents with either ethanol or methanol (depending on the extract) was used as a negative control and ethylenediaminetetraacetic acid (EDTA) as a reference chelating agent (in the 0.003–0.050 mg/mL concentration range). The iron-chelating activity was determined using Equation (2) and the results, presented as EC_50_ values (mg/mL), were calculated using a nonlinear regression analysis. Three independent experiments were performed and averaged.

### 4.12. Ferric Reducing Antioxidant Power Assay

The ferric reducing antioxidant power (FRAP) assay was carried out using a modified version of reported procedures [79]. The FRAP reagent was prepared by mixing acetate buffer (0.3 M, pH = 3.6), 2,4,6-tris(2-pyridyl)-*s*-triazine (TPTZ, 10 mM) in 40 mM HCl and FeCl_3_·6(H_2_O) (20 mM), in a ratio of 10:1:1 (*v*/*v*/*v*) and incubating at 37 °C before use. In a 96-well plate, 200 µL of FRAP reagent were added to 10 µL of each extract concentration (1:2 serial dilutions from the initial sample), in triplicate. After incubating at room temperature for 30 min (in the dark), absorbance was measured at 593 nm against a blank consisting of FRAP reagent with either ethanol or methanol (depending on the extract). Trolox (T) was used as a reference standard (in the 0.02–0.25 mg/mL concentration range) and the FRAP activity was expressed as mean ± standard deviation of three independent experiments as equivalents of Trolox per gram of extract (mg TE/g extract).

### 4.13. Cupric Ion Reducing Antioxidant Capacity Assay

The cupric ion reducing antioxidant capacity (CUPRAC) assay was carried out using a modified version of reported procedures [79]. A mixture of ammonium acetate buffer (1 M, pH = 7), neocuproine (7.5 mM) and CuCl_2_ (10 mM) was prepared in a ratio of 1:1:1 (*v*/*v*/*v*). In a 96-well plate, 150 µL of this mixture was added to 25 µL of each extract concentration (1:2 serial dilutions from the initial sample), in triplicate. After incubating at room temperature for 30 min (in the dark), absorbance was measured at 450 nm against a blank consisting of all the reagents with either ethanol or methanol (depending on the extract). Trolox was used as a reference standard (in the 0.02–0.25 mg/mL concentration range) and the CUPRAC activity was expressed as mean ± standard deviation of three independent experiments as equivalents of Trolox per gram of extract (mg TE/g extract).

### 4.14. Cholinesterase Inhibition

The inhibitory activity of the acetylcholinesterase enzyme was assayed using a modified version of literature-reported methods [80]. Briefly, in a 96-well plate, 160 µL of TRIS-HCl buffer (50 mM, pH = 8.0), 10 µL of 5,5′-dithiobis(2-nitrobenzoic acid) (DTNB, 2 mM), 10 µL of acetylthiocholine iodide (AChI, final concentration 0.68 mM) and 20 µL of each extract concentration (1:2 serial dilutions from the initial sample), were placed, in duplicate. The plate was incubated at room temperature, for 10 min, after which 20 µL of acetylcholinesterase (AChE) in Tris-HCl (0.45 U/mL) were added and the absorbances measured at 412 nm against a blank consisting of all the reagents except the enzyme. A mixture of Tris-HCl buffer, DTNB, AChI, AChE, and either ethanol or methanol (depending on the extract) was used as the control. Galantamine hydrobromide a known cholinesterase inhibitor used in the treatment of Alzheimer’s disease was used as a reference acetylcholinesterase inhibitor, and the EC_50_ value of 0.009 mg/mL was in accordance with the literature [81,82]. The enzyme inhibition activity (%) was determined using Equation (2) and the results, presented as EC_50_ values (mg/mL), were calculated using nonlinear regression analysis. Three independent experiments were performed and averaged.

### 4.15. Antimicrobial Activity Assay 

#### 4.15.1. Bacterial Strains and Culture Conditions

The strains used for the evaluation of the antimicrobial activity of the FBP extract were: *Escherichia coli* (ATCC 8739) and *Staphylococcus aureus* (ATCC 29213), purchased from the American Type Culture Collection; *Pseudomonas aeruginosa*, *Klebsiella oxytoca*, *Enterococcus faecalis*, *Escherichia coli* extended-spectrum beta-lactamase positive (ESβL), methicillin-resistant *Staphylococcus aureus* (MRSA) and *Klebsiella pneumoniae* producing carbapnemase (KPC), obtained from the Microbiology Laboratory, Division of Clinical Pathology, University Hospital of Coimbra. Intermediate cultures were prepared from the stock cultures in Tryptic Soy Agar. Fresh cultures (24 h) were used in all the experiments.

These strains were selected (Table S1 of Supplementary Material) according to their antibiotic susceptibility profile, determined using the VITEK 2 system (bioMérieux, France). Specific VITEK 2 AST cards were used for *Enterobacteriaceae*, non-fermenters, *Staphylococci*, and *Enterococci*.

#### 4.15.2. Microdilution Assay

The minimum inhibitory concentrations (MIC) of the FBP extract were determined using the broth microdilution method. The freeze-dried extract was solubilized in sterile distilled water until a concentration of 100 mg/mL was reached (in 96-well microtiter plates) and the assay was carried out in Mueller Hinton Broth (MHB) using serial dilutions between 0.195 and 50 mg/mL. The fresh (18–24 h) bacterial colonies were suspended in sterile 0.9% NaCl (*w/v*) to achieve turbidity of 0.5 MacFarland standard corresponding to ca. 1 × 10^8^ colony-forming units (CFU)/mL. The final concentration of each strain was adjusted to 5 × 10^5^ CFU, with 50 µL of bacterial suspension in MHB, in a final volume of 100 µL. Bacterial growth controls were prepared, without FBP extract (which was replaced by the same volume of sterile distilled water). MHB medium was used as a sterility control. All experiments were performed in triplicate.

The plates were covered with a sterile plate sealer, carefully mixed, and incubated at 37 °C for 24 h. After incubation, a *p*-iodonitrotetrazolium violet (INT) indicator was added (40 µL) to evaluate bacterial growth inhibition. The results were measured after 30 min at 37 ± 2 °C, by reading the absorbance at 570 nm. INT, initially yellow, changes to pink/purple when metabolized by living microorganisms. 

The percentage of viable cells was determined, according to Equation (4):

(4) $$\mathrm{Viable}\;\mathrm{cells}\;\left(\%\right)=\left[\frac{\mathrm{MOD}\;\mathrm{Extract}\;\mathrm{with}\;\mathrm{strain}-\mathrm{MOD}\;\mathrm{Extract}\;}{\mathrm{MOD}\;\mathrm{strain}-\mathrm{MOD}\;\mathrm{culture}\;\mathrm{medium}}\right]\times 100,$$

where the MOD extract with strain represents the mean of the optical density of the three readings obtained at 570 nm, after incubation of the extract at a given concentration with the MHB strain; MOD extract is the mean of the optical density of the three readings obtained at 570 nm after incubation of the extract at a given concentration in MHB; MOD strain is the mean of the optical density of the three readings obtained by at 570 nm after incubation of the strain in MHB; MOD culture medium represents the mean of the optical density of the three readings obtained at 570 nm after incubation of MHB culture medium.

Based on the antimicrobial effect of the FBP extract, the MIC values were determined for a zero percentage of the viable bacterial cells.

### 4.16. Spectroscopic Measurements

The FTIR–ATR spectra were acquired in the mid-infrared interval (400–4000 cm^−1^) using a Bruker Optics Vertex 70 FTIR spectrometer purged by CO_2_-free dry air and a Bruker Platinum ATR single reflection diamond accessory. A Ge on KBr substrate beamsplitter with a liquid nitrogen-cooled wide band mercury cadmium telluride detector was used. The spectra were corrected for the frequency dependence of the penetration depth of the electric field in ATR (considering a mean reflection index of 1.0, as previously used for other phytochemicals using the Opus 7.2 spectroscopy software) [13,53,83] and the three-term Blackman–Harris apodization function was applied. Each spectrum presented corresponded to the average of two measurements of 128 scans each, at a resolution of 2 cm^−1^.

The Raman spectra were recorded in the 450–3750 cm^−1^ range, in a WITec confocal Raman microscope system alpha300R. The device was coupled to an Ultra-High-Throughput-Spectrometer UHTS 300 Vis-NIR (300 mm focal length, 600 lines/mm blazed for 500 nm grating). The detection system was a thermoelectrically cooled CCD (charge-coupled device) camera with a Peltier cooling system reaching a temperature ≤−55 °C. A chip with 1650 × 200 pixels, front-illuminated with NIR/VIS anti-reflection coating and spectral resolution <0.8 cm^−1^/pixel. The excitation radiation was the 532 nm line of a diode laser, yielding 15–20 mW at the sample position. An objective Zeiss “Epiplan” 10X (NA 0.23; WD 11.1 mm) was employed. Five spectra were collected per extract sample (FBP, BSR, and BSO). Each spectrum was obtained with 5 accumulations and 10 s of exposure time without any previous treatment neither for the Raman nor for the FTIR-ATR acquisitions, being this fact one of the key aspects of both techniques. The spectra were acquired in different regions, being the result reproducible.

### 4.17. Statistical Analysis

The results were analyzed in GraphPad Prism 5 software (GraphPad Software, La Jolla, CA, USA) using one-way ANOVA (for three or more groups) followed by Tukey’s post hoc test for statistical comparison between the experimental data; *p*-values less than 0.05 were considered as significant, and these differences represented by different superscript letters in a column of results. The EC_50_ values were calculated for each extract fitting the results using nonlinear regression analysis, in sigmoidal dose–response curves (variable slope).

## 5. Conclusions

The use of natural products (NPs) for the development of new compounds with significant antioxidant and antimicrobial activity, for both nutraceutical and pharmaceutical use, is a research area of increasing interest.

The present work determined the vibrational and biological profiles of *C. album* berry pulp and seed methanolic extracts. The phytochemical characterization and evaluation of antioxidant potential confirmed that the seed is the most nutraceutical promising part of the berry, particularly the seed residue after oil extraction. Nevertheless, the *C. album* seed oil, currently extracted and characterized for the first time, presented a similar activity regarding the inhibition of the β-carotene bleaching and the ability to chelate copper ions. The pulp extract, in turn, showed a significant antimicrobial effect against several bacterial strains with clinical importance and different antibiotic susceptibility profiles, namely *E. faecalis*, *E. coli* ATCC 8739, *K. pneumoniae*, *S. aureus*, and *P. aeruginosa*.

The vibrational profile obtained by complementary FTIR and Raman spectroscopies revealed the presence of phenolic acids, polysaccharides, sugars, and triterpenoids in the pulp, high content of unsaturated fatty acids in the berry seed oil, and significant amounts of phenolic compounds and carotenoids in the seed residue which may justify its higher antioxidant capacity as compared to the other parts of the fruit.

In the light of the promising health-beneficial activities of this edible fruit, endogenous of the Portuguese coastline, further characterization at the molecular level is envisaged in order to assess the best way to exploit its full nutraceutical potential. It is mandatory to identify their major chemical components, for both the pulp and the seeds, and relate them to each of the diverse biological activities currently identified. FTIR and Raman vibrational spectroscopy are the methods of choice, in view of their high accuracy, simplicity (minimal amounts of sample and no preparation required), and non-destructive nature.

## Figures and Tables

**Table 1:** Total phenolic content (TPC, mg GAE/g extract), total flavonoid content (TFC, mg QCE/g extract), and total monomeric anthocyanin content (TMAC, mg C3GE/g extract) of pulp and seed extracts of *C. album* berries.

Extract	TPC	TFC	TMAC
FBP	9.9 ± 0.1 ^c^	1.7 ± 0.4 ^c^	0.06 ± 0.02 ^b^
BSR	41.0 ± 0.5 ^a^	19.6 ± 0.7 ^b^	4.6 ± 0.8 ^a^
BSO	17.6 ± 2.1 ^b^	79.6 ± 2.3 ^a^	1.6 ± 0.8 ^b^

FBP, fresh berries pulp; BSR, berries seed residue; BSO, berries seed oil; GAE, gallic acid equivalents; QCE, quercetin equivalents; C3GE, cyanidin-3-glucoside equivalents. Values represent the mean ± standard deviation of three independent experiments. For the same column, different superscript letters indicate significant differences (Tukey’s post hoc test, *p* < 0.05).

**Table 2:** Free radical scavenging activity (DPPH and ABTS) and inhibition of lipid peroxidation of pulp and seed extracts of *C. album* berries presented as EC_50_ values (mg/mL).

Extract/Standard	DPPH	ABTS	Lipid Peroxidation
FBP	3.1 ± 0.2 ^a^	>5	>5
BSR	0.15 ± 0.04 ^b^	1.09 ± 0.03	2.0 ± 0.2
BSO	>5	>5	>5
BHT	0.10 ± 0.03 ^b^	0.17 ± 0.03	0.009 ± 0.005

FBP, fresh berries pulp; BSR, berries seed residue; BSO, berries seed oil. BHT—reference antioxidant. Values represent the mean ± standard deviation of three independent experiments. For the same column, different superscript letters indicate significant differences (Tukey’s post hoc test, *p* < 0.05).

**Table 3:** Metal chelating activity (EC_50_, mg/mL) and ferric (FRAP) and cupric (CUPRAC) reducing powers (mg TE/g extract) of pulp and seed extracts of *C. album* berries.

Extract/Standard	Metal Chelating Activity	FRAP	CUPRAC
FBP	>5	12.0 ± 0.7 ^b^	24.7 ± 2.0 ^c^
BSR	4.2 ± 0.2	54.7 ± 4.9 ^a^	146.6 ± 5.9 ^a^
BSO	>5	6.8 ± 1.1 ^b^	127.3 ± 2.4 ^b^
EDTA	0.015 ± 0	-	-

FBP, fresh berries pulp; BSR, berries seed residue; BSO, berries seed oil; TE, Trolox equivalents. EDTA—reference chelating agent. Values represent the mean ± standard deviation of three independent experiments. For the same column, different superscript letters indicate significant differences (Tukey’s post hoc test, *p* < 0.05).

**Table 4:** Minimum inhibitory activity (MIC) of the FBP extract against bacterial strains.

Bacterial Strains	MIC of the FBP Extract (mg/mL)
*Escherichia coli* ATCC 8739	6.25
*Staphylococcus aureus* ATCC 29213	12.5
*Pseudomonas aeruginosa*	12.5
*Klebsiella oxytoca*	25
*Enterococcus faecalis*	3.125
*Escherichia coli* ESβL	50
Methicillin-resistant *Staphylococcus aureus*	12.5
*Klebsiella pneumoniae* KPC	6.25

## Supplementary Materials

The following are available online at https://www.mdpi.com/article/10.3390/plants10091761/s1, Figure S1: Raman spectrum of the BSR extract. The grey marks highlight the polyene characteristic signals., Table S1: Characterization of the bacterial strains used in this study (origin and antibiotic susceptibility profile).
