# Supplementary material for: Novel Insights into Corema album Berries: Vibrational Profile and Biological Activity
Source: Plants (Basel). 2021 Aug 25;10(9):1761. doi: 10.3390/plants10091761 (PMC8470319; doi:10.3390/plants10091761)
Supplement: Supplementary file 1 [file plants-10-01761-s001.zip › plants-1335367-supplementary.pdf]

Supplementary Material

Novel insights into *Corema album* Berries: Vibrational Profile and Biological Activity

Joana Marques [1*], Daniel Martin [1], Ana M. Amado [1], Viktoriya Lysenko [2], Nádia Osório [1,2], Luís A. E. Batista de Carvalho [1], Maria Paula M. Marques [1,3], Maria João Barroca [1,4], Aida Moreira da Silva [1,4]

1 Unidade de I&D Química-Física Molecular, Department of Chemistry, University of Coimbra, 3004-535 Coimbra, Portugal; marques.jt@uc.pt (J.M.); dmfernandez@uc.pt (D.M.); ama@uc.pt (A.M.A.); nadia.osorio@estescoimbra.pt (N.O.); labc@ci.uc.pt (L.A.E.B.d.C.); pmc@ci.uc.pt (M.P.M.M.); mjbarroca@esac.pt (M.J.B.); aidams@esac.pt (A.M.d.S.)
2 College of Health Technology of Coimbra, Polytechnic Institute of Coimbra, S. Martinho do Bispo, 3046-854 Coimbra, Portugal; v.lysenko2@health.gov.je (V.L.)
3 Department of Life Sciences, University of Coimbra, 3000-456 Coimbra, Portugal
4 Polytechnic of Coimbra, Coimbra Agriculture School, Bencanta, 3045-601 Coimbra, Portugal
* Correspondence: marques.jt@uc.pt

Table S1. Characterization of the bacterial strains used along this study (origin and antibiotic susceptibility profile).

Bacterial strain	Origin	Antibiotic Susceptibility Profile
Pseudomonas aeruginosa	urine	R: TIC<br>S: CTZ, GM, CIP, TZP, MEM, LVX, IPM, AK, ATM, TM, FEP
Klebsiella oxytoca	wound exudate non-surgical	R: AMP, FOS<br>S: AMC, CXM–Sodium, GM, CIP, SXT, TZP, CTX, CTZ, MEM, AK, CXM-axetil, CL, FEP, ETP
Enterococcus faecalis	urine	R: SXT, QD<br>S: AMP, LVX, VA, TEC, NIF, STR, GEN, IPM, LZD, TGC
Escherichia coli ESβL	blood	R: AMP, AMC, CXM-sodium, CTX, CTZ, GM, CIP, SXT, FEP<br>S: MEM, AK, TZP
Methicillin-resistant *S. aureus*	sputum	S: TET, RD, TEC, LZD, GEN, FAD, MUP, SXT<br>R: E, CLI, LVX, OX, FOX
Klebsiella pneumoniae KPC	urine	R: AMP, AMC, CXM-sodium, CTX, CTZ, MEM, GM, CIP, SXT, TZP, FEP, ETP<br>S: CL, TGC CIM

R – resistant, S – susceptible, AK – amikacin, AMX – amoxicillin, AMC – amoxicillin+clavulanic acid, AMP – ampicillin, ATM – aztreonam, FEP – cefepime, FOX – cefoxitin, CTX – cefotaxime, CXM – cefuroxime, CTZ – ceftazidime, CRO – ceftriaxone, CIP – ciprofloxacin, CL – colistin, ETP – ertapenem, FOS – fosfomycin, GM - gentamicin, GEN – gentamicin (high load), IPM – imipenem, LVX – levofloxacin, LZD – linezolid, MEM – meropenem, NIF – nitrofurantoin, OX – oxacillin, QD – quinupristin/dalfopristin, STR – streptomycin (high load), TZP – piperacillin/tazobactam, TEC – teicoplanin, TIC – ticarcillin, TGC – tigecycline, TM – tobramycin, SXT – trimethoprim-sulfamethoxazole, VA – vancomycin, TET – tetracycline, RD – rifampicin, FAD – fusidic acid, MUP – mupirocin, E – erythromycin, CLI – clindamycin.

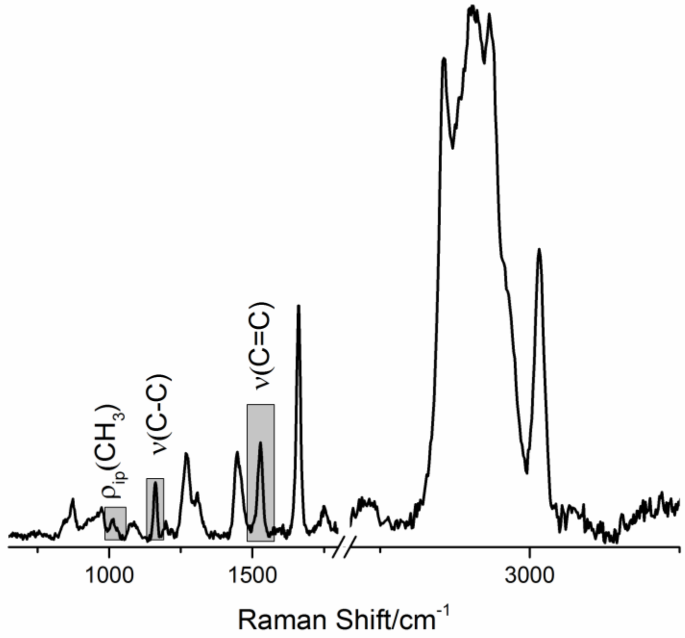

Figure S1. Raman spectrum of the BSR extract. The grey marks highlight the polyene characteristic signals.
